# Cell cycle accumulation of the proliferating cell nuclear antigen PCN-1 transitions from continuous in the adult germline to intermittent in the early embryo of *C. elegans*

**DOI:** 10.1186/s12861-018-0171-7

**Published:** 2018-05-30

**Authors:** Zuzana Kocsisova, Kerry Kornfeld, Tim Schedl

**Affiliations:** 1 Department of Developmental Biology, Washington University in St. Louis, 660 S. Euclid Ave, St. Louis, MO 63108 USA; 2 Department of Genetics, Washington University in St. Louis, 660 S. Euclid Ave, St. Louis, MO 63108 USA

**Keywords:** PCNA, S-phase, *C. elegans*, Germline, Cell cycle, *pcn-1*

## Abstract

**Background:**

The proliferating cell nuclear antigen (PCNA or PCN-1 in *C. elegans*), an essential processivity factor for DNA polymerase δ, has been widely used as a marker of S-phase. In *C. elegans* early embryos, PCN-1 accumulation is cyclic, localizing to the nucleus during S-phase and the cytoplasm during the rest of the cell cycle. The *C. elegans* larval and adult germline is an important model systems for studying cell cycle regulation, and it was observed that the cell cycle regulator cyclin E (CYE-1 in *C. elegans*) displays a non-cyclic, continuous accumulation pattern in this tissue. The accumulation pattern of PCN-1 has not been well defined in the larval and adult germline, and the objective of this study was to determine if the accumulation pattern is cyclic, as in other cells and organisms, or continuous, similar to cyclin E.

**Results:**

To study the larval and adult germline accumulation of PCN-1 expressed from its native locus, we used CRISPR/Cas9 technology to engineer a novel allele of *pcn-1* that encodes an epitope-tagged protein. S-phase nuclei were labeled using EdU nucleotide incorporation, and FLAG::PCN-1 was detected by antibody staining. All progenitor zone nuclei, including those that were not in S-phase (as they were negative for EdU staining) showed PCN-1 accumulation, indicating that PCN-1 accumulated during all cell cycle phases in the germline progenitor zone. The same result was observed with a GFP::PCN-1 fusion protein expressed from a transgene. *pcn-1* loss-of-function mutations were analyzed, and *pcn-1* was necessary for robust fertility and embryonic development.

**Conclusions:**

In the *C. elegans* early embryo as well as other organisms, PCN-1 accumulates in nuclei only during S-phase. By contrast, in the progenitor zone of the germline of *C. elegans*, PCN-1 accumulated in nuclei during all cell cycle stages. This pattern is similar to accumulation pattern of cyclin E. These observations support the model that mitotic cell cycle regulation in the germline stem and progenitor cells is distinct from somatic cells, as it does not heavily rely on cyclic accumulation of classic cell cycle proteins.

## Background

Regulation of cell proliferation is key to a wide range of health concerns, from infertility to wound healing and cancer. Like Goldilocks, every tissue in every living organism must find the level of cell division that is “just right” in order to survive, develop, and pass on genetic information. The cell cycle coordinates duplication and segregation of both genetic and cellular material to two daughter cells. Cell cycle regulators change abundance or localization at different stages of DNA replication and cell division. For example, cyclin E accumulates during the G1-S-phase transition. This pattern is generally conserved between multiple eukaryotic kingdoms of life. However, the adult *C. elegans* germline accumulates cyclin E in all cell cycle phases, suggesting that this tissue may utilize distinctive mechanisms of cell cycle control [1].

**Fig. 1:**
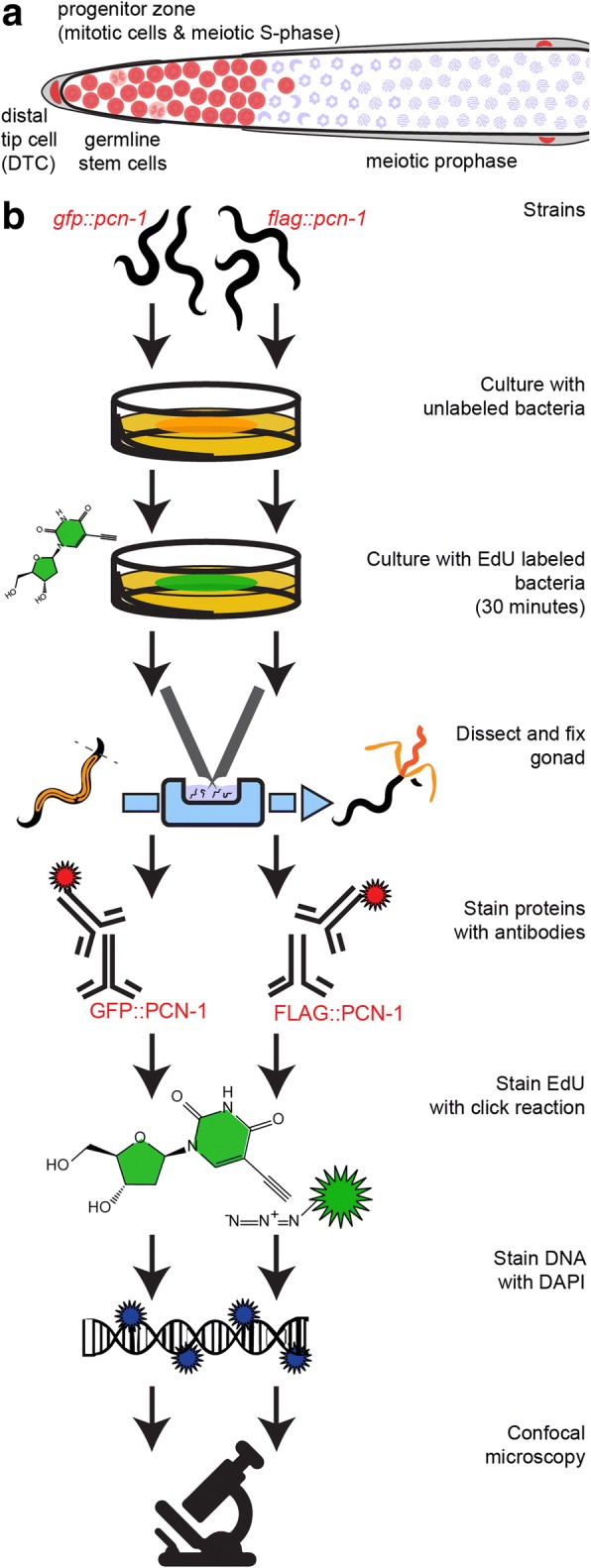
Diagram of distal *C. elegans* germline and experimental workflow. **a** The syncytial distal progenitor zone (highlighted in red based on WAPL-1 antibody staining) contains mitotically cycling stem and progenitor cells and cells in meiotic S-phase. The distal tip cell (DTC) provides GLP-1 signal (Notch ligand) to maintain the stem cell fate of these cells. As cells migrate away from the DTC, they exit the progenitor zone and enter meiotic prophase. **b** Workflow used to assay the relationship between PCN-1 accumulation and S-phase (EdU labeling) in the *C. elegans* germline

The distal *C. elegans* hermaphrodite germline contains the only stem cells in the adult *C. elegans* (Fig. 1a). The somatic distal tip cell (DTC) surrounds the syncytial distal-most nuclei and provides the niche to maintain these stem cells in their proliferative fate. The ~ 20 cell-diameter long distal region, which includes the mitotically cycling germline stem and progenitor cells and meiotic S-phase cells but not cells in meiotic prophase, is called the progenitor zone [2–5]. As germ cells move away from the DTC, the cells finish the mitotic cell cycle, enter the meiotic cell cycle, undergo meiotic S-phase, and enter prophase I of meiosis [3].

The proliferating cell nuclear antigen (PCNA or PCN-1 in *C. elegans*) is an essential processivity factor for DNA polymerase δ (Reviewed in [6]). As its name suggests, PCNA was identified by its nuclear accumulation in proliferating cells [7]. In mammals, antibody recognition of PCNA is an often-used technique to identify cells in S-phase [8]. Brauchle et al. [9] established PCN-1 as a useful in vivo marker of S-phase in *C. elegans* early embryos, a stage when the cell cycle involves only negligible gap phases. In transgenic worms that express a green fluorescent protein GFP::PCN-1 fusion protein under the control of the *pie-1* promoter, GFP::PCN-1 localizes to the nucleus during S-phase, resulting in a bright fluorescent signal. At nuclear envelope breakdown (beginning of mitosis), GFP::PCN-1 localizes to the cytoplasm, resulting in a diffuse, low level signal. Similarly, GFP::PCNA protein injected into the gonad serves as a marker of S-phase in both *C. elegans* pronuclei and early embryonic divisions [10].

For studies of cell cycle dynamics in the *C. elegans* adult germline, labeling with nucleotide analogs such as 5-ethynyl-2′-deoxyuridine (EdU) or 5-bromo-2′-deoxyuridine (BrdU) has been the gold standard to identify S-phase [1, 2]. However, these chemicals must enter *C. elegans* by feeding or soaking, which limits the utility of this approach. For example, some older adult animals fail to label with EdU following a short (e.g. 30 min) exposure, which might reflect defects in ingestion and/or transport of EdU ([11], our unpublished observations). To clarify the relationships between PCN-1 accumulation and nucleotide analog incorporation as markers of S-phase, we developed methods to combine these two approaches.

To visualize PCN-1 in the adult germline, we used CRISPR/Cas9 genome editing to modify the native *pcn-1* locus to encode a 3xFLAG epitope at the N-terminus of PCN-1*.* Surprisingly, FLAG::PCN-1 accumulated in all nuclei in the germline progenitor zone. By contrast, a short pulse of EdU revealed that only about half of these nuclei were in S-phase. These results suggest that the accumulation and localization of PCN-1 is regulated differently in the *C. elegans* germline, where it is present in all progenitor zone nuclei, compared to the embryo, where it is restricted to nuclei in S-phase. Furthermore, we demonstrated that *pcn-1* is an essential gene in *C. elegans* necessary for both adult fertility and embryonic development. These results extend the understanding of the accumulation and function of PCN-1 in *C. elegans*, an important model system for studies of germline development and cell cycle regulation.

## Methods

*C. elegans* strains were maintained at 20 °C on 6 cm dishes of nematode growth media (NGM) seeded with OP50 *E. coli* [12]. The wild-type strain was Bristol N2. The GZ264 strain contains the GFP::PCN-1 transgene (*isIs17*) inserted at an undefined location in the genome, and it is wild-type at the *pcn-1* locus [9]. To engineer a FLAG epitope tag into the endogenous *pcn-1* locus, we used the co-CRISPR approach [13]. The injection mix contained Cas9-expressing pDD162 (gift from Mike Nonet) at 50 ng/μl, *dpy-10* guide plasmid (pMN3153) at 20 ng/μl, *dpy-10(cn64)* ssDNA repair template AFZF827 at 500 nM, *pcn-1* guide plasmids (pZK21 and pZK23) at 40 ng/μl for each plasmid, and a ssDNA repair template (ZK071 containing the *3xflag* sequence) at 600 nM. We injected the gonads of P0 wild-type hermaphrodites, selected F1 progeny that displayed the Rol phenotype, and used PCR to select animals with the desired genotype. Two independently derived strains contained the same insertion of the 22 amino acid coding region for the 3xFLAG epitope after the initial methionine codon of *pcn-1*. We named these alleles *pcn-1(am315)* and *pcn-1(am316)* (strains WU1764 and WU1765, respectively). We used a GFP-tagged balancer chromosome to maintain the strain *pcn-1(am315)/nT1g* due to the observed maternal-effect lethal phenotype.

To confirm expression of the full-length FLAG::PCN-1 product, we performed a Western blot. In brief, animals were washed from one 12 cm NGM dish and rinsed 4 times with M9 buffer. Protease inhibitor (Thermo Scientific Pierce Protease Inhibitor Mini Tablets Cat# 88665) was used at 2× listed concentration in the second two washes. An equal volume of 2× lysis-and-loading buffer (100 mM TrisHCl pH 6.8, 200 mM DTT, 4% SDS, 0.2% Bromophenol Blue, 20% Glycerol) was added, and the samples were frozen at − 80 °C overnight. The samples were microwaved, boiled, sonicated in an ice bath, and boiled again. Samples (1.5, 7.5, or 15 uL) and a size reference (7uL, AccuRuler Prestained Protein Ladder LambdaBio Cat# G02101) were loaded into a pre-cast gel (BioRad Mini-PROTEAN TGX 4–20% polyacrylamide gel Cat#456–1093) in 1X Tris/ Glycine/ SDS buffer and run at 180 V for ~ 1 h in an ice bath. Samples were transferred to activated membrane (Millipore Immobilon-P) in 1X Tris/ Glycine/ 20%Methanol buffer at 350 mA for ~ 1 h in an ice bath. The membrane was incubated in blocking buffer (5% non-fat milk in 1X PBS + 0.5% Tween-20) for ~ 1 h at room temperature with agitation. Primary mouse-anti-FLAG antibody (Sigma M2 F-1804) was diluted 1:10,000 in blocking buffer and applied overnight at 4 °C with agitation. The membrane was rinsed 3× with PBSTw. Secondary anti-mouse HRP antibody (Cell Signaling) was diluted 1:2000 in blocking buffer and applied for ~ 1 h at room temperature with agitation. The membrane was rinsed 3× with PBSTw, incubated with substrate (ThermoScientific SuperSignal West Femto Maximum Sensitivity Substrate) for ~ 1 min, and imaged on a ChemiDoc imaging system. Protein size was estimated from conceptually translated DNA sequence using the Science Gateway Protein Molecular Weight Calculator (http://www.sciencegateway.org/tools/proteinmw.htm).

To visualize both FLAG::PCN-1 accumulation and cells in S-phase, we cultured fourth larval stage (L4) and young adult progeny of heterozygous *pcn-1(am315/+)* parent animals on EdU-labeled *E. coli* MG1693 for 30 min and promptly dissected [1]. Animals were fixed with 3% paraformaldehyde for 10 min and post-fixed in 100% methanol at − 20 °C overnight. Primary mouse-anti-FLAG antibody (Sigma M2 F-1804) was diluted 1:1000 and rabbit-anti-WAPL antibody (Novus 49300002) was diluted 1:20000 in 30% goat serum and applied overnight. Secondary goat-anti-mouse-Alexa-647 antibody and secondary goat-anti-rabbit-Alexa-594 antibody (Life Technologies) were diluted 1:400 in 30% goat serum and applied for 4 h. The Click-iT EdU Fluor 488 reaction was performed according to manufacturer instructions (ThermoFisher Cat#C10350). Animals were mounted in Vectashield + DAPI on agarose pads and imaged using a 63×/1.4NA Plan Apo oil immersion objective on a Zeiss Observer Z1 inverted microscope equipped with a Perkin-Elmer Ultraview Vox spinning disc confocal system using Volocity software. Exposure times were 150 ms for EdU, 50 ms for FLAG::PCN-1, and 500 ms for DAPI. Images were processed with Fiji [14] and Adobe Illustrator. GFP::PCN-1 was visualized as described above, except primary rabbit-anti-GFP (gift from Swathi Arur) and secondary goat-anti-rabbit-Alexa-594 were used (Fig. 1b).

To quantify fluorescence intensity, we used the region of interest ROI and Measure tools in Fiji. In brief, a cell was selected in a single plane based on DAPI channel and assigned to S-phase or gap phase based on the presence or absence of EdU. A single ROI was drawn to surround the DAPI signal, and the mean intensity of DAPI, EdU, and PCN-1 was obtained for the ROI. Background intensities of all three channels were very low and comparable, and therefore no background subtraction was performed.

Statistical analyses were performed in R [15] using R studio [16] and ggplot2 [17]. Data were compared using an ANOVA with Tukey’s Honest Significant Difference post-hoc. All error bars shown in figures represent the mean +/− standard deviation.

We determined the genotype and phenotype of animals descended from P0 heterozygous *pcn-1(am315)/+* or *pcn-1(am316)/+* hermaphrodites. F1 animals were cultured individually for 3 to 4 days from egg. Each animal was observed for viability and fertility. Animals which deposited no eggs on the substrate were dissected to allow unlaid F2 eggs to develop. One to 2 days later, F2 progeny were examined for viability. Genotype was established by performing PCR analysis on individual F1 animals. Hermaphrodites were defined as “fertile” if they deposited one or more eggs on the agar surface that hatched and developed into larvae. Animals were defined as “sterile” if they deposited no eggs on the agar surface, or “dead eggs” if all deposited eggs failed to hatch.

Heterozygous animals were fertile and did not display obvious defects when cultured in standard laboratory conditions. The GFP-tagged balancer chromosome *nT1g* was used to track the *pcn-1* genotype in a larger number of animals. For brood size assays, P0 *pcn-1(am315)/nT1g* males were mated to *fog-2(q71)* females. The non-green F1 progeny of this cross, *pcn-1(am315/+); fog-2(q71/+)* were synchronized at the mid-L4 stage, isolated to individual NGM dishes, and moved to fresh dishes every 24 h. Parent animals which fled the dish or died of matricidal hatching were excluded. The F2 progeny were counted ~ 2 days later until the cessation of reproduction. *fog-2(q71/+)* served as the control. For survival assays (egg-to-larva and egg-to-adult), the identical crosses were performed, and F1 animals were allowed to lay eggs for several hours. These eggs were transferred to a fresh dish and counted 24 h later, the unhatched eggs were counted to estimate the number of larvae, and the adults were counted 48 h later.

## Results

### PCN-1 accumulated during all cell cycle phases in the germline progenitor zone

**Fig. 2:**
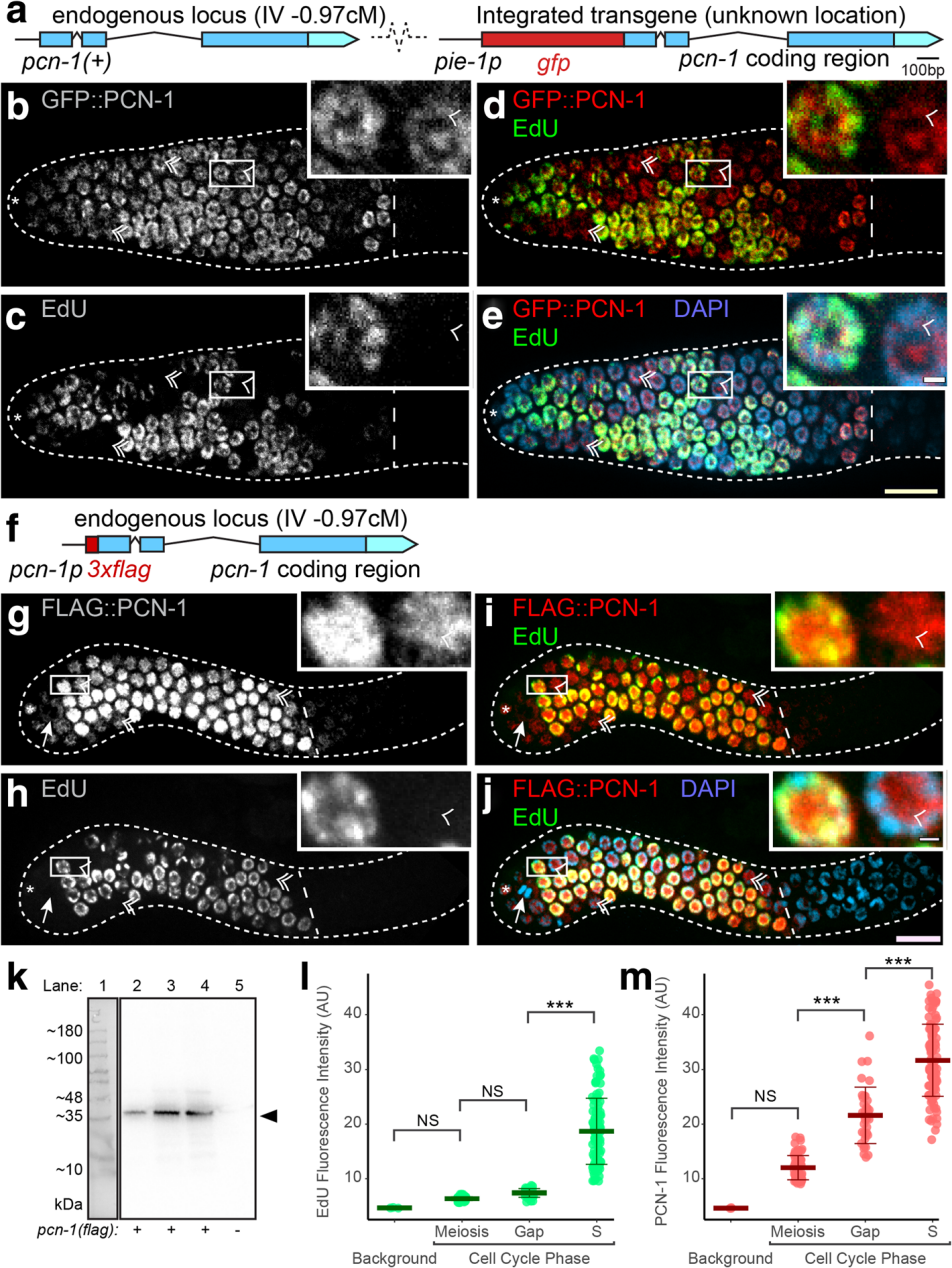
PCN-1 accumulated during all cell cycle phases in the germline progenitor zone. **a** A diagram of a portion of the genome of the GZ264 strain that contains the GFP::PCN-1 transgene (*isIs17*) inserted at an undefined genomic location. The *pie-1* promoter drives transcription in the germline and is fused to the coding regions of GFP (red) and PCN-1(blue). Boxes indicate exons with light shading for the 3’ UTR, straight lines indicate promoters, and peaked lines indicate introns. The strain contains an intact *pcn-1* locus on chromosome IV. Scale bar is 100 bp. **b**-**e** Confocal microscope images of a hermaphrodite gonad at the adult stage. The dotted white line outlines the gonad, and the dashed white line marks the end of the progenitor zone. Note that not all nuclei are in the focal plane. The asterisk marks the position of the distal tip cell. The inset shows an enlargement of the region outlined by a white rectangle - scale bars are in panel E. Main scale bar is 10um, inset scale bar is 1um. Single arrowheads (in inset) and double arrowheads (outside of inset) mark nuclei that were negative for EdU staining (in gap phase) and positive for GFP::PCN-1 accumulation. White or red mark GFP::PCN-1 accumulation visualized by antibody staining (**b**, **d**, **e**). White or green mark EdU staining visualized by click chemistry (**c**, **d**, **e**). Yellow indicates overlap (**d**). Blue marks DAPI staining for DNA (**e**). GFP::PCN-1 accumulated in all progenitor zone nuclei in an adult hermaphrodite, whereas only about half the nuclei were positive for EdU staining. **f** A diagram of the *pcn-1(am315)* genomic locus. The endogenous *pcn-1* promoter drives transcription and is fused to the coding regions of 3xFLAG epitope tag (red) inserted in-frame immediately after the ATG start codon to create an N-terminally tagged fusion protein expressed from the endogenous locus. **g**-**j** Confocal microscope images of a *pcn-1(am315)*/+ hermaphrodite gonad at the late L4 stage. The dotted white line outlines the gonad, and the dashed white line marks the end of the progenitor zone. Note that not all nuclei are in the focal plane. The asterisk marks the position of the distal tip cell. The inset shows an enlargement of the region outlined by a white rectangle – scale bars are in panel J. Main scale bar is 10um, inset scale bar is 1um. Single and double arrowheads mark nuclei that were negative for EdU staining (in gap phase) and positive for FLAG::PCN-1 accumulation. The arrow points to an anaphase nucleus. Main scale bar is 10um, inset scale bar is 1um. White or red mark FLAG::PCN-1 visualized by antibody staining (**g**, **i**, **j**); it accumulated in all progenitor zone nuclei. White or green mark EdU staining visualized by click chemistry (**h**, **i**, **j**). Yellow indicates overlap (**i**). Blue marks DAPI staining for DNA (**j**). Longer exposures of panels B and G show that all nuclei are PCN-1 positive. Longer exposures of panels D and I show that a 30 min EdU pulse, which marks S-phase nuclei, stains about 50% of progenitor zone nuclei. Antibody staining for WAPL-1 was used to define progenitor zone cells. **k** Western blot probed with anti-FLAG antibody. Lane 1: Markers, with sizes indicated in kDa. Lanes 2, 3, and 4: 1.5ul, 7.5ul, and 15ul protein lysate from *pcn-1(am315flag)/nT1g* animals, respectively. Lane 5: 7.5ul protein lysate from wild-type animals. The Western blot shows an ~ 35 kDa band specific to the lanes containing *pcn-1(am315flag)* (arrowhead on right), which corresponds well with the expected 31.75 kDa size. **l**, **m** Quantification of fluorescence intensity of EdU (**l**) and FLAG::PCN-1 (**m**). Background values were obtained from image regions without tissue. Nuclei were selected in the DAPI channel and assigned to phases as follows: nuclei without WAPL-1 signal were assigned to meiosis (*n* = 73), nuclei with both WAPL-1 and EdU signal were assigned to S-phase (*n* = 104), and nuclei with WAPL-1 but without EdU signal were assigned to gap phases (*n* = 36). ANOVA with Tukey’s Honest Significant Difference post-hoc was used to compare the mean fluorescence intensity of nuclei; NS indicates *P* > 0.01, * *P* < 0.01, ** *P* < .001, *** *P* < .0001. The distributions of gap and S-phase EdU intensity were mutually exclusive; the FLAG::PCN-1 distributions of gap and S-phase display 74% overlap

PCN-1 accumulation has been monitored using an integrated transgene that encodes a GFP::PCN-1 fusion protein (Fig. 2a). In *C. elegans* early embryos, PCN-1 accumulates during S-phase but not during M phase, indicating that it is regulated during the cell cycle [9, 10]. Thus, we anticipated that PCN-1 would display a similar accumulation pattern in the larval and adult germline. To test this prediction, we monitored S-phase nuclei using a short, 30 min pulse of EdU and we monitored PCN-1 expression with an anti-GFP antibody to detect the GFP::PCN-1 fusion protein (Fig. 1b). The nucleotide analog EdU is used as a selective marker of DNA synthesis as it is incorporated into DNA during S-phase. In the germline, S-phase occurs only in progenitor zone cells, which include the stem cells (Fig. 1a). Consistent with previous publications, approximately 50% of progenitor zone nuclei were EdU positive, indicating that these cells were in S-phase (Fig. 2c) [1, 2, 18]. By contrast, all progenitor zone nuclei were GFP::PCN-1 positive, irrespective of their cell cycle stage (Fig. 2b). Thus, about half the progenitor zone nuclei were positive for both EdU and GFP::PCN-1, indicating that cells in S-phase display nuclear accumulation of PCN-1 (Fig. 2d). However, about half the progenitor zone nuclei were negative for EdU and positive for GFP::PCN-1, indicating nuclei that were not in S-phase, and presumably were in G2, M, or the short G1 phase, also accumulated PCN-1 (Fig. 2d, e). It is notable that the GFP fluorescence signal in the germline displayed extremely low intensity, and we could only detect the GFP::PCN-1 fusion protein with long exposure times for GFP fluorescence in live animals or after staining with anti-GFP antibodies.

Two features of the transgenic strain raise the possibility that the accumulation pattern of the GFP::PCN-1 fusion protein may not accurately reflect the accumulation pattern of endogenous PCN-1; (1) fusion to the large GFP protein may affect the accumulation pattern or (2) the transgene uses the heterologous *pie-1* promoter and is integrated at a random site in the genome, not the endogenous locus (Fig. 2a). To address these concerns, we used the CRISPR/Cas9 technique to edit the *C. elegans* genome. We chose the small FLAG epitope tag, reasoning that it is less likely to influence protein behavior than GFP. However, we cannot exclude the possibility that the FLAG tagged protein does not behave identically to the endogenous protein. We inserted coding sequence for three copies of the FLAG epitope tag at the N-terminus of PCN-1 into the native *pcn-1* locus (Fig. 2f). Two independently derived strains with the identical insertion were generated, and we named these alleles *pcn-1(am315)* and *pcn-1(am316)*. The presence of a full-length FLAG::PCN-1 fusion protein was confirmed by performing an anti-FLAG Western blot (Fig. 2k).

To monitor the expression pattern, we dissected and immunostained animals using an antibody directed against the FLAG epitope. The antibody staining was performed on the progeny of *pcn-1(am315/+)* animals, and thus included animals of three genotypes: *pcn-1(am315/am315), pcn-1(am315/+)* and *pcn-1(+/+)*. While the genotype of each individual was not determined using molecular approaches, we observed that the majority of animals showed the same pattern of FLAG::PCN-1 in the distal germline. Specifically, FLAG::PCN-1 was detected in all cells in the progenitor zone (Fig. 2g). We interpreted these animals as a mixture of *pcn-1(am315/am315)* and *pcn-1(am315/+).* Thus, it is likely that *pcn-1(am315/am315)* and *pcn-1(am315/+)* animals have a similar pattern of expression*.* A minority of animals displayed no staining*.* We interpreted these animals as *pcn-1(+/+)*.

Several features of the expression pattern were notable. In metaphase and anaphase nuclei (identified by DAPI morphology), FLAG::PCN-1 signal appeared somewhat diffuse and to surround the chromatin (Fig. 2g). By contrast, FLAG::PCN-1 signal in G1, S, and G2 nuclei overlapped with both chromatin and the nucleolus. Similar to the results described above with GFP::PCN-1, we detected multiple examples of nuclei in gap phase which displayed no EdU incorporation but did display nuclear FLAG::PCN-1 accumulation (Fig. 2g–j). The same accumulation pattern was observed with both *pcn-1(am315)* and *pcn-1(am316)* strains. PCNA has been reported to show a punctate nuclear pattern in S-phase of mammalian cells [19, 20]. We did not observe punctate FLAG::PCN-1 staining; it is possible that harsh fixation conditions contribute to the lack of punctate staining.

To explore the relationship between PCN-1 expression and S-phase in detail, we quantified the intensity of FLAG::PCN-1 and EdU signal (following a 30 min labeling) in individual distal germline nuclei (Fig. 2l–m). Meiotic prophase cells that are not replicating DNA were used to establish the level of background noise. For EdU, the current gold-standard for S-phase labeling in *C. elegans*, progenitor zone nuclei either displayed very low intensity signal, similar to background, or substantial signal; we assigned these nuclei to gap phase or S-phase, respectively. By contrast, all of the progenitor zone nuclei displayed FLAG::PCN-1 staining that was higher than background, indicating that all of these cells accumulate the protein. Thus, the presence of FLAG::PCN-1 cannot be used to reliably determine if a cell is in S-phase or gap phase. Cells in S-phase had a higher average level of FLAG::PCN-1 staining than cells in gap phase, but the distributions were highly overlapping: 71% of the true S-phase nuclei and 81% of the true gap phase nuclei (74% of total nuclei) were in the ambiguous range of FLAG::PCN-1 intensity (Fig. 2l–m). Thus, FLAG::PCN-1 appears to be continually present in nuclei, although it accumulates to higher levels during S phase.

**Fig. 3:**
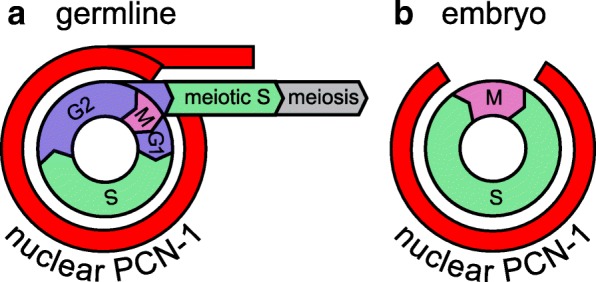
The adult germline and embryo show distinct patterns of PCN-1 nuclear accumulation. The inner circle represents the stages of the cell cycle; DNA replication occurs in S-phase (green), mitosis occurs in M phase (pink), and these are separated by G2 and a very short G1 phase (blue) [1]. Germline stem cells progress to meiotic S-phase and meiosis (gray). The outer circle represents nuclear accumulation of PCN-1 (red). Germline stem cells in *C. elegans* display a more typical mitotic cell cycle with gap phases and also display constant (non-cycling) accumulation of several proteins, including PCN-1 and CYE-1, which have been reported to display cell cycle regulated accumulation in other tissues and organisms (**a**) [1, 22]. By contrast, early embryos in *C. elegans* display a simplified cell cycle (S,M), and PCN-1, like several other cell cycle proteins, show nuclear accumulation only during S-phase (**b**) [9, 10]

In the *C. elegans* early embryo, GFP::PCN-1 shows nuclear accumulation only during S-phase [9, 10]). A limitation of our use of FLAG::PCN-1 is that visualization cannot be performed in live embryos. Thus, our results do not establish the expression pattern of FLAG::PCN-1 in early embryos. By contrast to embryos, our results indicate that in the L4 and adult germline progenitor zone, PCN-1 accumulates throughout the cell cycle and its presence cannot be used as a reliable marker of S-phase (Fig. 3). It is likely that germline PCN-1 is regulated by mechanisms other than degradation/translation and nuclear localization.

### *pcn-1* was necessary for robust fertility and embryonic development

The GZ264 strain that contains the GFP::PCN-1 transgene (*isIs17*) and is wild-type at the *pcn-1* locus (Fig. 2a) is viable and fertile. Compared to wild-type animals, this strain displayed abnormal, tortuous intestinal morphology after only 2–3 days of adulthood and a higher rate of vulval extrusion. The self-fertile brood size was not different from wild type, but the lifespan was slightly reduced (GZ264 16.6 ± 5.0 *n* = 60; N2 20.5 ± 5.1 *n* = 120, Log-Rank Test *P* = .0000019). These phenotypes could be caused by expression of the GFP::PCN-1 fusion protein, a deleterious effect of insertion of the transgene into the genome at a site of functional significance, or additional background effects.

To determine how the insertion of the sequence encoding the FLAG epitope at the *pcn-1* locus affected gene function, we analyzed heterozygous and homozygous mutant animals. To rigorously quantify the penetrance of phenotypes, we determined the genotype and phenotype of 31 animals descended from *pcn-1(am315)/+* hermaphrodites (Table 1a). Genotype was established by performing PCR analysis on individual animals. Heterozygous animals were fertile and did not display obvious defects when cultured in standard laboratory conditions. Animals homozygous for either *pcn-1(am315)* or *pcn-1(am316)* that descended from heterozygous P0 hermaphrodites developed to adulthood and displayed sterility or maternal-effect embryonic lethality (Table 1a). These homozygous mutant animals produced few F2 homozygous embryos. Some F1 homozygous mutant hermaphrodites did not lay any embryos and displayed F2 embryos retained inside the uterus. Other F1 homozygous mutant hermaphrodites deposited a few embryos on the substrate, but these embryos failed to develop. This result was confirmed in an independent experiment (without PCR genotyping) examining 60 F1 progeny of heterozygous P0 animals; 15 of these animals (25%) produced no viable progeny. These results indicate that both *pcn-1(am315)* and *pcn-1(am316)* are loss-of-function alleles, and *pcn-1* function is necessary to promote hermaphrodite fertility. In addition, *pcn-1* appears to have a function early in development that can be maternally rescued, since *pcn-1* homozygous mutants derived from heterozygous hermaphrodites develop to adulthood, whereas *pcn-1* homozygous mutants derived from *pcn-1* homozygous hermaphrodites displayed embryonic lethality.

**Table 1:** Genetic analysis of *pcn-1* fertility. **A)** Thirty-one self-progeny of heterozygous *pcn-1(am315flag)/+* hermaphrodites were analyzed by PCR of single animals to determine genotype after egg-laying was complete. Full genotypes: *pcn-1(+/+)*, *pcn-1(am315/+)*, and *pcn-1(am315/am315)*. Hermaphrodites were scored as “fertile” if they deposited one or more eggs on the agar surface that hatched and developed into larvae. Animals were scored as “sterile” if they deposited no eggs on the agar surface, or if all deposited eggs failed to hatch. **B)** The percent of eggs that generate larvae and adults was determined for *pcn-1(+/+); fog-2(q71/+)* and *pcn-1(am315flag/+); fog-2(q71/+)* hermaphrodites, as described in the methods. n refers to total number of eggs analyzed. A chi-square test was used to compare the larval and adult survival. The total self-fertile brood size was determined as described in the methods. ANOVA was used to compare the brood size. Mean ± s.d. n refers to total number of parental worms analyzed. NS indicates *P* > 0.01, * *P* < 0.01, ** *P* < .001, *** *P* < .0001

A	Genotype		
	*pcn-1 (+/+)*	*pcn-1 (flag/+)*	*pcn-1 (flag/flag)*
Fertile	7	17	0
Sterile	0	0	7
B	Parental Genotype		
	*pcn-1 (+/+)*	*pcn-1 (flag/+)*	
Larval survival (%)	98.1 (*n* = 376)	98.1 (*n* = 324)	NS
Adult survival (%)	96.0 (*n* = 376)	95.7 (*n* = 324)	NS
Total Brood Size	305.4 ± 35.5 (*n* = 15)	332.5 ± 35.7 (*n* = 21)	NS

To determine if the *pcn-1* mutation causes a dominant effect, we analyzed survival and fertility in heterozygous animals under standard laboratory conditions. Progeny of *pcn-1(am315/+)* heterozygous mutants displayed the same rates of egg hatching (98%) and survival to adulthood (96%) as progeny of *pcn-1(+/+)* animals (Table 1b). The self-fertile brood size of *pcn-1(am315/+)* hermaphrodites was not significantly reduced compared to *pcn-1(+/+)* hermaphrodites (Table 1b). These results indicate that the FLAG::PCN-1 protein does not interfere with the endogenous protein function and *pcn-1(lf)* mutations are recessive for the fertility and survival phenotypes.

## Discussion

We set out to engineer an endogenous non-feeding marker for S-phase in the *C. elegans* germline. We chose the gene PCN-1 for this purpose, as it has been used as an S-phase marker in mammals and in the *C. elegans* embryo. Both the GFP::PCN-1 transgene and our CRISPR-engineered FLAG::PCN-1 allele showed that PCN-1 localizes to the nucleus in all cells in the progenitor zone, regardless of whether the cells are in S-phase or gap phase. Therefore, PCN-1 was not a useful marker of S-phase under our conditions. The localization of PCN-1 to all nuclei in the progenitor zone was similar to the pattern of cyclin E1 (CYE-1) in the *C. elegans* germline [1]. While this result means PCN-1 is not an S-phase marker, it reveals that the *C. elegans* germline likely regulates S-phase in an unusual way.

No previous publications have described the *pcn-1* loss-of-function phenotype. The *C. elegans* database (WormBase WS257) lists a deletion allele, *pcn-1(tm3241)* that is predicted to be a loss-of-function mutation*.* A strain that contains this allele displays a lethal or sterile phenotype, consistent with our findings; however, the *pcn-1(tm3241)* strain has not been backcrossed, and these phenotypes might reflect background mutations [21]. We found *pcn-1(am315)* animals showed a maternal-effect embryonic lethal phenotype. While it is unclear whether *am315* is a hypomorph or null allele, our results show that *pcn-1* is necessary for fertility.

## Conclusions

We established the nuclear accumulation pattern of PCN-1 in the larval and adult germline by analyzing FLAG::PCN-1 encoded by the native locus and GFP::PCN-1 encoded by an integrated transgene. Both fusion proteins displayed similar patterns and accumulated in all nuclei in the progenitor zone. Significantly, nuclei that were negative for EdU staining, and thus not in S-phase, displayed nuclear PCN-1 accumulation (Fig. 3). Similar results were previously observed for cyclin E accumulation (CYE-1 in *C. elegans*) [1]. These observations reinforce that mitotic cell cycle regulation in the germline stem and progenitor cells is distinct from somatic cells [1]. The *C. elegans* proliferating cell nuclear antigen homolog *pcn-1* was necessary for robust fertility and embryonic development.

## Abbreviations

*C. elegans*: *Caenorhabditis elegans*
GFP: Green fluorescent protein
PCNA: Proliferating cell nuclear antigen
PFA: Paraformaldehyde

## References

[1] Fox PM, Vought VE, Hanazawa M, Lee M-H, Maine EM, Schedl T (2011). Cyclin E and CDK-2 regulate proliferative cell fate and cell cycle progression in the C. elegans germline. Development.

[2] Crittenden SL, Leonhard KA, Byrd DT, Kimble J (2006). Cellular analyses of the mitotic region in the Caenorhabditis elegans adult germ line. Mol Biol Cell.

[3] Fox PM, Schedl T (2015). Analysis of germline stem cell differentiation following loss of GLP-1 notch activity in Caenorhabditis elegans. Genetics.

[4] Hansen D, Schedl T (2013). Stem cell proliferation versus meiotic fate decision in Caenorhabditis elegans. Adv Exp Med Biol.

[5] Pazdernik N, Schedl T (2013). Introduction to germ cell development in caenorhabditis elegans. Adv Exp Med Biol.

[6] Moldovan GL, Pfander B, Jentsch S (2007). PCNA, the maestro of the replication fork. Cell.

[7] Miyachi K, Fritzler MJ, Tan EM (1978). Autoantibody to a nuclear antigen in proliferating cells. J Immunol.

[8] Galand P, Degraef C (1989). Cyclin/PCNA immunostaining as an alternative to tritiated thymidine pulse labelling for marking S phase cells in paraffin sections from animal and human tissues. Cell Prolif.

[9] Brauchle M, Baumer K, Gönczy P (2003). Differential activation of the DNA replication checkpoint contributes to asynchrony of cell division in C. elegans embryos. Curr Biol.

[10] Kisielewska J, Lu P, Whitaker M (2005). GFP-PCNA as an S-phase marker in embryos during the first and subsequent cell cycles. Biol Cell.

[11] Cinquin A, Chiang M, Paz A, Hallman S, Yuan O, Vysniauskaite I (2016). Intermittent stem cell cycling balances self-renewal and senescence of the C. elegans germ line. PLoS Genet.

[12] Brenner S (1974). The genetics of Caenorhabditis elegans. Genetics.

[13] Arribere JA, Bell RT, Fu BXH, Artiles KL, Hartman PS, Fire AZ (2014). Efficient marker-free recovery of custom genetic modifications with CRISPR/Cas9 in Caenorhabditis elegans. Genetics.

[14] Schindelin J, Arganda-Carreras I, Frise E, Kaynig V, Longair M, Pietzsch T (2012). Fiji: an open-source platform for biological-image analysis. Nat Methods.

[15] R Core Team (2013). R: a language and environment for statistical computing.

[16] RStudio Team (2015). RStudio: integrated development environment for R.

[17] Wickham H (2009). ggplot2: elegant graphics for data analysis.

[18] Jaramillo-Lambert A, Ellefson M, Villeneuve AM, Engebrecht J (2007). Differential timing of S phases, X chromosome replication, and meiotic prophase in the C. elegans germ line. Dev Biol.

[19] Hahn AT, Jones JT, Meyer T (2009). Quantitative analysis of cell cycle phase durations and PC12 differentiation using fluorescent biosensors. Cell Cycle.

[20] Leonhardt H, Rahn H-P, Weinzierl P, Sporbert A, Cremer T, Zink D (2000). Dynamics of DNA replication factories in living cells. J Cell Biol.

[21] Barstead R, Moulder G, Cobb B, Frazee S, Henthorn D, Holmes J, Jerebie D, Landsdale M, Osborn J, Pritchett C, Robertson J, Rummage J, Stokes E, Vishwanathan M, Mitani S, Gengyo-Ando K, Funatsu OR, Consortium, T. C. E. D. M (2012). Large-scale screening for targeted knockouts in the Caenorhabditis elegans genome. G3.

[22] Leonardi E, Girlando S, Serio G, Mauri FA, Perrone G, Scampini S (1992). PCNA and Ki67 expression in breast carcinoma: correlations with clinical and biological variables. J Clin Pathol.

